# Respiratory muscle training: a bibliometric analysis of 60 years’ multidisciplinary journey

**DOI:** 10.1186/s12938-023-01103-0

**Published:** 2023-05-22

**Authors:** Muhammad Imran Ramli, Nur Azah Hamzaid, Julia Patrick Engkasan, Juliana Usman

**Affiliations:** 1 Department of Biomedical Engineering, Faculty of Engineering, Universiti Malaya, 50603 Kuala Lumpur, Malaysia; 2 Biomechatronics and Neuroprosthetics Laboratory, Department of Biomedical Engineering, Faculty of Engineering, Universiti Malaya, 50603 Kuala Lumpur, Malaysia; 3 Department of Rehabilitation Medicine, Faculty of Medicine, Universiti Malaya, 50603 Kuala Lumpur, Malaysia

**Keywords:** Bibliometric, Multidisciplinary, Research trend, Ventilatory muscle training, Visualisation map

## Abstract

**Background:**

Over the decades, many publications have established respiratory muscle training (RMT) as an effective way in improving respiratory dysfunction in multiple populations. The aim of the paper is to determine the trend of research and multidisciplinary collaboration in publications related to RMT over the last 6 decades. The authors also sought to chart the advancement of RMT among people with spinal cord injury (SCI) over the last 60 years.

**Methods:**

Bibliometric analysis was made, including the publications’ profiles, citation analysis and research trends of the relevant literature over the last 60 years. Publications from all time frames were retrieved from Scopus database. A subgroup analysis of publications pertinent to people with SCI was also made.

**Results:**

Research on RMT has been steadily increasing over the last 6 decades and across geographical locations. While medicine continues to dominate the research on RMT, this topic also continues to attract researchers and publications from other areas such as engineering, computer science and social science over the last 10 years. Research collaboration between authors in different backgrounds was observed since 2006. Source titles from non-medical backgrounds have also published articles pertinent to RMT. Among people with SCI, researchers utilised a wide range of technology from simple spirometers to electromyography in both intervention and outcome measures. With various types of interventions implemented, RMT generally improves pulmonary function and respiratory muscle strength among people with SCI.

**Conclusions:**

While research on RMT has been steadily increasing over the last 6 decades, more collaborations are encouraged in the future to produce more impactful and beneficial research on people who suffer from respiratory disorders.

## Introduction

Respiratory muscle training (RMT) involves specific exercise that requires either inspiration, expiration or both to stimulate the respiratory muscles. Just like any other skeletal muscles, RMT overloaded the fibers in the respiratory muscles by increasing the intensity, time and frequency of the training itself [1]. RMT not only enhances the respiratory muscles’ strength and resistance, but it also may improve their endurance [2]. The development of blood lactate concentration during exercise, sympathetic activation, and muscle fatigue among healthy participants can also be reduced through RMT [3–5].

Miller et al. was one of the earliest available publications to mention training in breathing among their participants, albeit less vigorous, not specific and established as current publications [6]. Miller et al. also still acknowledged that breathing exercise increased work capacity and tolerance, allowing a more efficient breathing pattern feasible among people with severe respiratory insufficiency due to pulmonary emphysema [6]. Meanwhile, Gould and Okamura was one of the first available studies that incorporated RMT among their targeted population [7]. In this case, Gould and Okamura found out that through proper respiratory training, professional singers increased their ventilatory capacity, thus allowing a more efficient way in voice production [7].

Since then, many more literatures on RMT have been published to improve respiratory dysfunction and allow a more effective style of breathing [2, 8–15]. Other than healthy individuals [2], many other published review articles from various populations had reported enhanced strength and/or endurance after RMT. This includes people with spinal cord injury (SCI) [8], stroke [9], multiple sclerosis [10], Parkinson’s disease [11], chronic heart failure [12], hypoxia [13], acute respiratory condition [14], and chronic obstructive pulmonary disease (COPD) [15].

RMT can be categoriezed in many ways and forms, depending on the methods utilized during the training. For instance, resistive inspiratory muscle training and expiratory muscle training requires the patient to breathe through a small diameter resistor that involves a one way valve system [16]. This technique increases the ventilatory load by limiting the available airflow [17]. Normocapnic hyperpnoea involves a device that requires the patient to fill and empty a breathing bag, which is in 30% to 40% of the forced vital capacity [18, 19]. This technique also trains both inspiratory and expiratory respiratory muscles simultaneously. Isocapnic hyperpnoea requires the patient to maintain high target levels of ventilation for up to 30 min [20]. Therapeutic singing requires the patient to be in an extensive, vigorous and forceful training in order to get significant results on the targeted population [18].

In order to prescribe and monitor the RMT intervention more specifically, quantitative measurements were increasingly adopted throughout the years. Intervention tools has become more technically advanced and the quantification of the patients’ breathing performance has also been more robust and accurate. This has created a trend of multidisciplinary field involvement as technological interventions became more commonly adapted. Specialist and therapist are increasingly employing methods and techniques that are able to differentiate and discriminate user performance more precisely. Over the years, while medical specialist are actively reporting studies related to RMT, experts from other fields such as engineering, neuroscience, health professionals, biochemistry, genetics, and molecular biology has played significant role in pushing the boundaries of RMT intervention.

Up to this day, there is no publication that have reported the bibliometric analysis of literatures related to RMT. Bibliometric analysis is important in demonstrating the productivity of research through the publication trends over a certain period of time. It is also useful in illustrating the history and evolution of research over time. In our case, bibliometric analysis is also used in examining the trend of multidisciplinary among researchers from different background over the years. Other than medicine, we want to see whether researchers from different background and areas have collaborated with their research related to RMT over the last 6 decades. In this study, this bibliometric analysis is important in finding out the current research trends related to RMT since the earliest research published in the 60 s. Besides, the authors were also interested in finding out the progress of RMT among people with SCI over the last 60 years. This includes the types of interventions, main outcome measures or parameters, every devices or tools used in the training, and the effectiveness of the RMT.

Consequently, the first objective of this review is to determine the research and publications trends in RMT since the first literature pertinent to the topic was published. Other than document profiles and the citation analysis, the trend and expansion of the relevant publications was also made in this paper. Besides, the research collaboration or multidisciplinary trends in RMT over the last 6 decades was also sought after in this review. The final objective is to find out the progress of RMT among people with SCI over the last 60 years, as an illustration of the multidisciplinary involvement in the field of RMT. This includes the interventions, main outcome measures, and effectiveness of RMT in this population. Randomized controlled trial (RCT) studies were included in this section of review as they are often regarded as the most reliable evidence on the effectiveness of interventions. Due to the processes behind RCT studies, it reduces bias and minimises risk of factors that may influence the results.

Ergo, these are the research questions pertinent to this bibliometric analysis:

1. What are the progress of RMT studies over the last 6 decades?
2. When do researchers from non-medical backgrounds start involved in RMT?
3. What types of interventions and outcomes are used in studies involving people with spinal cord injury?

## Results

The literature search resulted in a total of 367 documents. No records were removed from the original search.

### Documents profiles

The database search revealed that the majority of the documents were scientific articles (Table 1) and came from journal (Table 2). English language was the dominant medium used in the documents with Chinese came in second (Table 3). Meanwhile, medicine dominated the subject area of the literatures identified in this analysis (Table 4). The percentages of each document type, source type, languages and subject area is derived from their total publications (TP), which is 367 documents. In the case of Table 4, some literatures were categorized into multiple categories by Scopus. Therefore, the total publications (TP) in this table does not sum up to 367. The percentage also does not sum up to 100%.

**Table 1:** Document type

Document type	Total publications (TP)	Percentage (%)
Article	265	72.21
Review	41	11.17
Conference paper	21	5.72
Letter	16	4.36
Note	10	2.72
Book chapter	5	1.36
Short survey	4	1.09
Editorial	2	0.54
Erratum	2	0.54
Book	1	0.27
Total	367	100.00

**Table 2:** Source type

Source type	Total publications (TP)	Percentage (%)
Journal	349	95.10%
Conference proceeding	12	3.27%
Book	5	1.36%
Book series	1	0.27%
Total	367	100.00

**Table 3:** Languages

Language	Total publications (TP)	Percentage (%)
English	303	80.59
Chinese	12	3.19
German	11	2.93
Portuguese	11	2.93
Japanese	10	2.66
French	8	2.13
Italian	6	1.60
Spanish	5	1.33
Russian	3	0.80
Moldovan	2	0.54
Czech	1	0.27
Danish	1	0.27
Hebrew	1	0.27
Polish	1	0.27
Romanian	1	0.27
Total	367	100.00

**Table 4:** Top 15 subject area

Subject area	Total publications (TP)	Percentage (%)
Medicine	307	83.65
Health professions	94	25.61
Biochemistry, genetics and molecular biology	45	12.26
Neuroscience	33	8.99
Nursing	16	4.36
Engineering	14	3.81
Computer science	8	2.18
Social sciences	7	1.91
Immunology and microbiology	6	1.63
Pharmacology, toxicology and pharmaceutics	5	1.36
Chemical engineering	4	1.09
Environmental science	3	0.82
Physics and astronomy	3	0.82

Some literature are categorized into more than one subject area by Scopus.

### Research trends

**Fig. 1:**
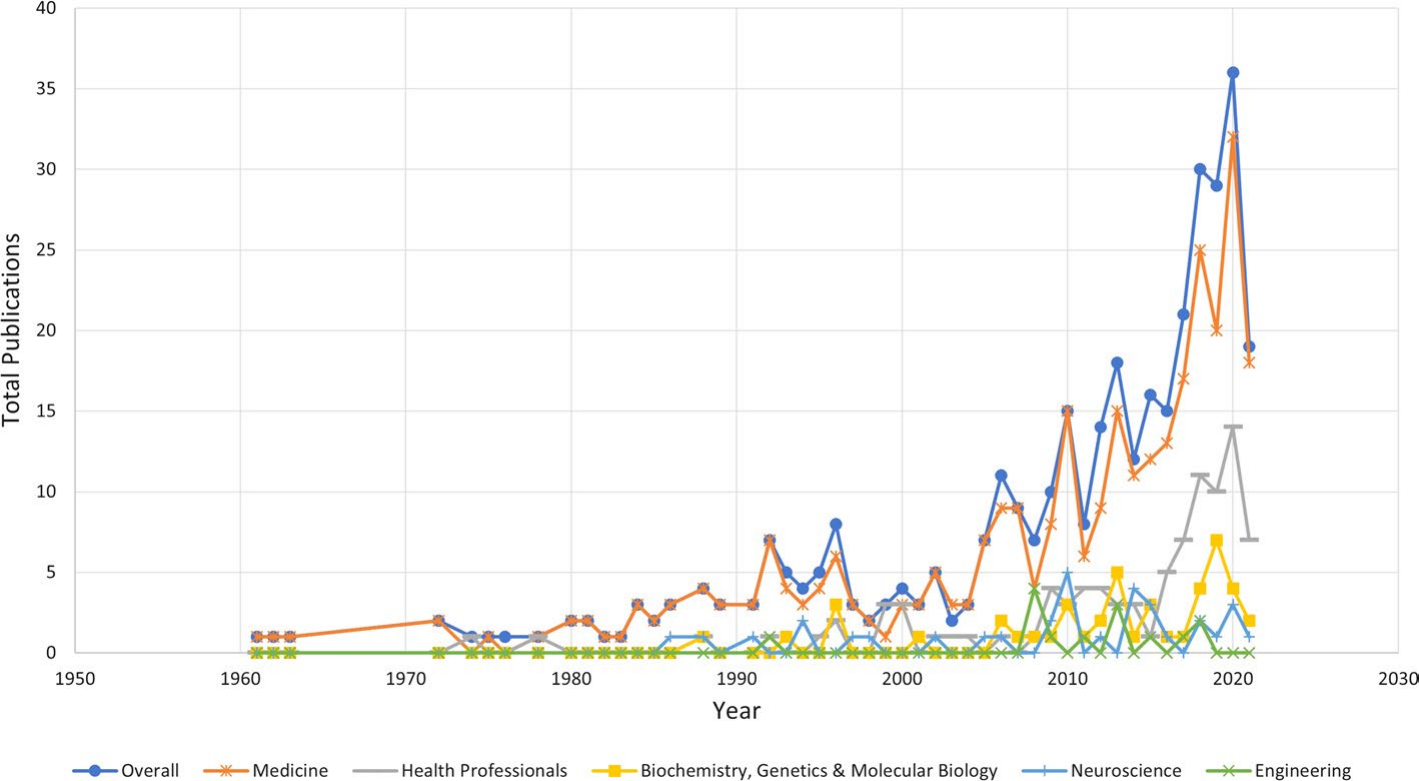
Total Publications and Citations by Year and Subject Area

**Fig. 2:**
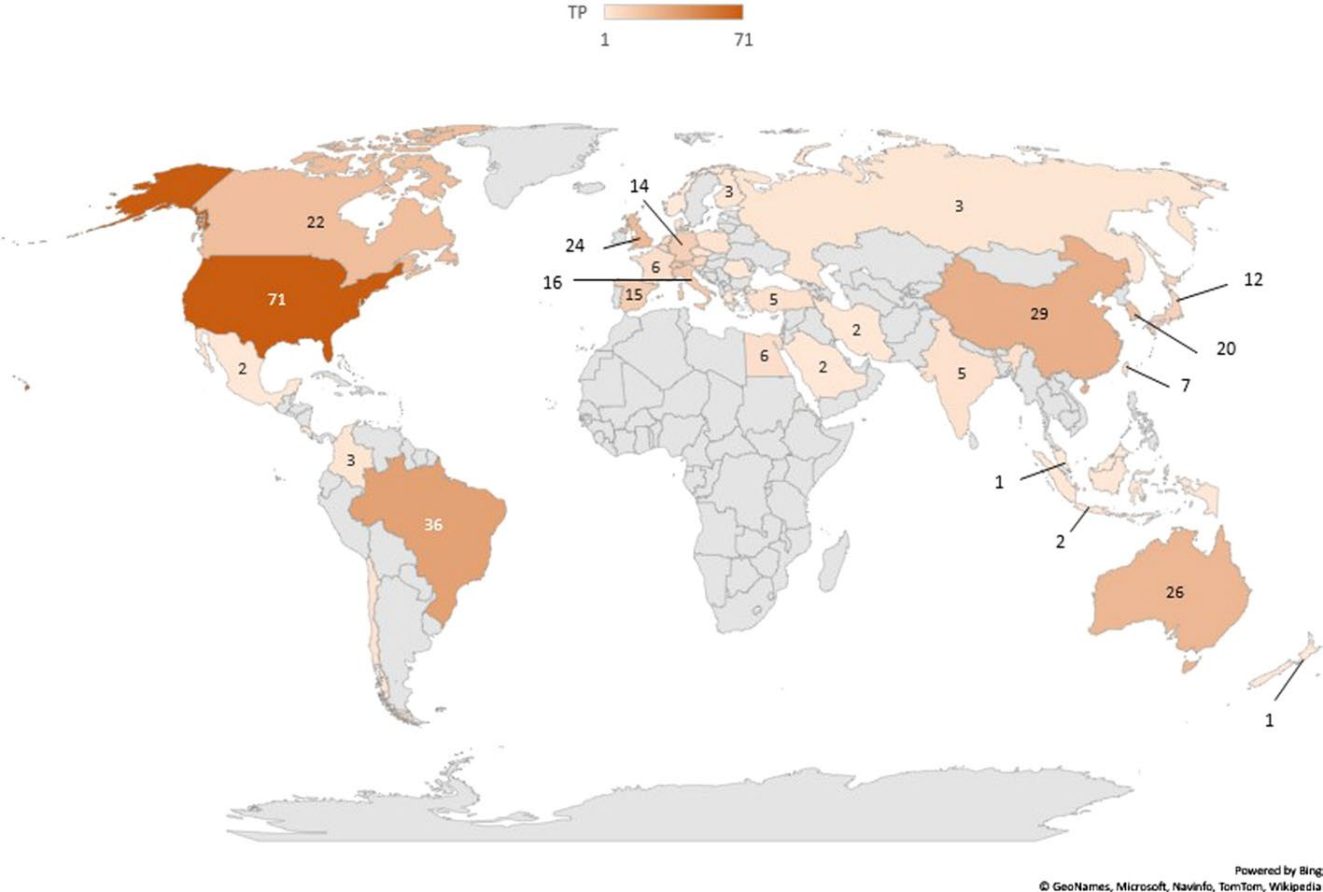
Geographical Distribution of Total Number of Publications

The database search revealed that there was a steady increase in the total publications (TP) over the last 10 years (Table 5). Figure 1 also shows the similar trend of increase in TP since the year 2004 in several subject areas, including medicine and health professionals. Based on the geographical distribution of the TP in Fig. 2, Europe was where most of the publications originated from (30% of the TP), while North America (26%) and Asia (24%) came in second and third, respectively.

**Table 5:** Number of publications and citations over the last 10 years

Year	TP	NCP	TC	C/P	C/CP	*h*	*g*
2021	21	3	3	0.14	1.00	1	1
2020	37	20	55	1.49	2.75	4	5
2019	29	20	88	3.03	4.40	5	8
2018	30	24	145	4.83	6.04	8	10
2017	21	15	115	5.48	7.67	8	10
2016	15	15	182	12.13	12.13	8	13
2015	16	12	147	9.19	12.25	8	12
2014	12	11	206	17.17	18.73	9	12
2013	18	11	370	20.56	33.64	9	18
2012	14	13	246	17.57	18.92	6	14
2011	8	6	205	25.63	34.17	6	8

TP = total number of publications; NCP = number of cited publications; TC = total citations; C/P = average citations per publication; C/CP = average citations per cited publication; h = h-index; and g = g-index

Table 6 shows the top ten most productive authors according to their total publication (TP) with the earliest in Loveridge et al. [16] and the latest one in Soumyashree and Kaur [33] and Boswell-Ruys et al. [34]. Author David R. Pendergast lead the rank based on the TP, NCP, h- and g-indices. Meanwhile, Christina M. Spengler lead the rank based on the TC, C/P and C/CP. The Archives Of Physical Medicine And Rehabilitation was reported to be most active source title based on both the TP and TC of the journal (Table 7).

**Table 6:** 10 most productive authors

Author’s Name	Affiliation		Country	TP	NCP	TC	C/P	C/CP	*h*	*g*
	Centre	Institution								
Pendergast, D.R	Department of Physiology and Biophysics	University at Buffalo, The State University of New York	United States	10	10	191	19.10	19.10	7	10
Lundgren, C.E.G	Center for Research and Education in Special Environments	University at Buffalo, The State University of New York	United States	7	7	164	23.43	23.43	6	7
Spengler, C.M	Zurich Center for Integrative Human Physiology	University of Zurich	Switzer-land	7	6	296	42.29	49.33	4	7
Crisp, K.D	Department of Head and Neck Surgery and Communication Sciences	Duke University School of Medicine	United States	5	3	38	7.60	12.67	2	5
Jaenisch, R.B	Department of Physiotherapy and Rehabilitation	Universidade Federal de Santa Maria	Brazil	5	4	63	12.60	15.75	4	5
Jones, H.N	Department of Head and Neck Surgery and Communication Sciences	Duke University School of Medicine	United States	5	3	38	7.60	12.67	2	5
Kravitz, R.M	Division of Pediatric Pulmonary and Sleep Medicine	Duke University School of Medicine	United States	5	3	38	7.60	12.67	2	5
Nascimento, L.R	Department of Physiotherapy	Universidade Federal de Minas Gerais	Brazil	5	4	78	15.60	19.50	3	5
Ray, A.D	Department of Rehabilitation Sciences	Roswell Park Cancer Institute	United States	5	4	71	14.20	17.75	4	5
Teixeira-Salmela, L.F	Department of Physiotherapy	Universidade Federal de Minas Gerais	Brazil	5	4	78	15.60	19.50	3	5

TP = total number of publications; NCP = number of cited publications; TC = total citations; C/P = average citations per publication; C/CP = average citations per cited publication; h = h-index; and g = g-index

**Table 7:** 10 most active source title

Source title	TP	TC	Publisher	Cite Score	SJR 2020	SNIP 2020
Archives Of Physical Medicine And Rehabilitation	12	296	Elsevier	5.7	1.305	1.728
Respiratory Physiology And Neurobiology	7	96	Elsevier	3.3	0.629	0.792
Revista Neurociencias	7	15	Universidade Federal de Sao Paulo	0.3	0.13	0.164
Clinical Rehabilitation	6	163	SAGE	4.9	1.15	1.696
Cochrane Database Of Systematic Reviews	6	102	Wiley-Blackwell	7.1	1.319	1.723
Journal Of Voice	6	8	Elsevier	3.7	0.772	1.891
Medical Physics	6	0	Wiley-Blackwell	6.1	1.473	1.555
Chest	5	171	Elsevier	10.3	2.647	2.764
European Journal Of Applied Physiology	5	197	Springer Nature	4.8	1.05	1.188
Thorax	5	61	BMJ Publishing Group	13.5	3.083	2.902

TP = total number of publications; TC = total citations; SJR = scientific journal ranking; SNIP = source normalized impact per paper

### Citation analysis

Overall, the combined citations metrics of the every literature related to RMT are summarized in Table 8. Meanwhile, Mereles et al. was the top cited article that relate to RMT with a total of 463 citations and an average of 30 citations per year (Table 9). It is also worth to note that three out of ten publications in Table 9 were review papers. The earliest literature review was published in 1992.

**Table 8:** Citations’ metrics

Metrics	Data
Papers	367
Number of citations	4951
Years	60
Citations per year	82.52
Citations per paper	13.49
Citations per author	1301.11
Papers per author	123.1
Authors per paper	4.28
h index	35
g index	61

**Table 9:** 10 Highly cited articles

No	Authors	Title	Year	Cites	Cites per year
1	Mereles et al	Exercise and respiratory training improve exercise capacity and quality of life in patients with severe chronic pulmonary hypertension	2006	463	30.87
2	Smith et al	Respiratory muscle training in chronic airflow limitation: A meta-analysis	1992	210	7.24
3	Mancini et al	Benefit of selective respiratory muscle training on exercise capacity in patients with chronic congestive heart failure	1995	188	7.23
4	Illi et al	Effect of respiratory muscle training on exercise performance in healthy individuals: A systematic review and meta-analysis	2012	162	18.0
5	Gosselink et al	Respiratory muscle weakness and respiratory muscle training in severely disabled multiple sclerosis patients	2000	121	5.76
6	Grünig et al	Effect of exercise and respiratory training on clinical progression and survival in patients with severe chronic pulmonary hypertension	2011	118	11.8
7	McConnell and Romer	Respiratory muscle training in healthy humans: Resolving the controversy	2004	108	6.35
8	Hajghanbari et al	Effects of respiratory muscle training on performance in athletes: A systematic review with meta-analyses	2013	101	12.63
9	Nomori et al	Preoperative respiratory muscle training: Assessment in thoracic surgery patients with special reference to postoperative pulmonary complications	1994	97	3.59
10	Sonetti et al	Effects of respiratory muscle training versus placebo on endurance exercise performance	2001	96	4.80

### Visualisation map

**Fig. 3:**
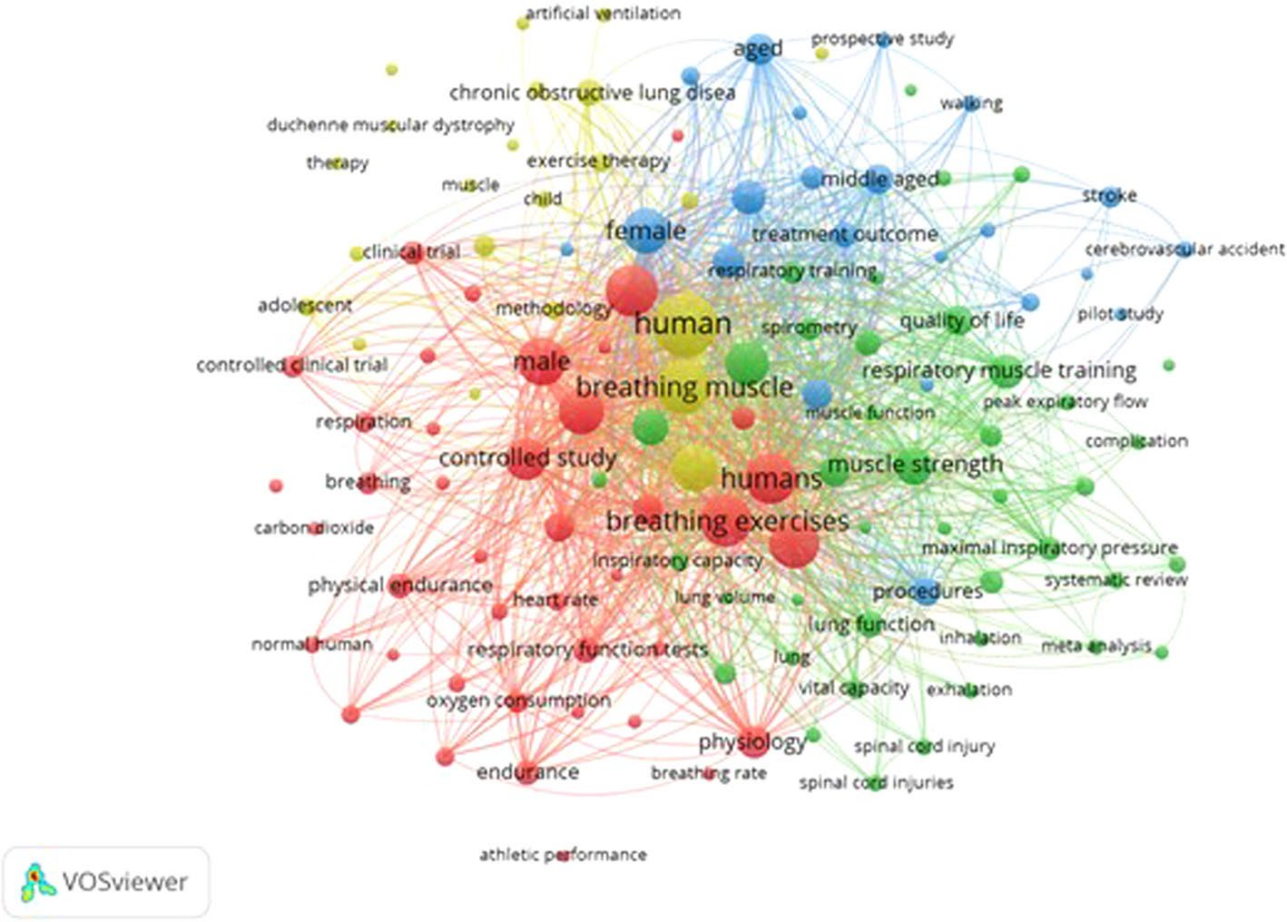
Network visualisation map of the author and index keywords. Full counting, minimum number of occurrences of a keyword: 10

Figure 3 reveals the network visualisation map of author and index keywords co-occurrence. Note that a minimum of ten occurrences of a keyword was set up before map was retrieved from VOSviewer software. The figure shows the relatedness of the keywords gathered from all of the literatures and grouped into four clusters (with four different colours).

**Fig. 4:**
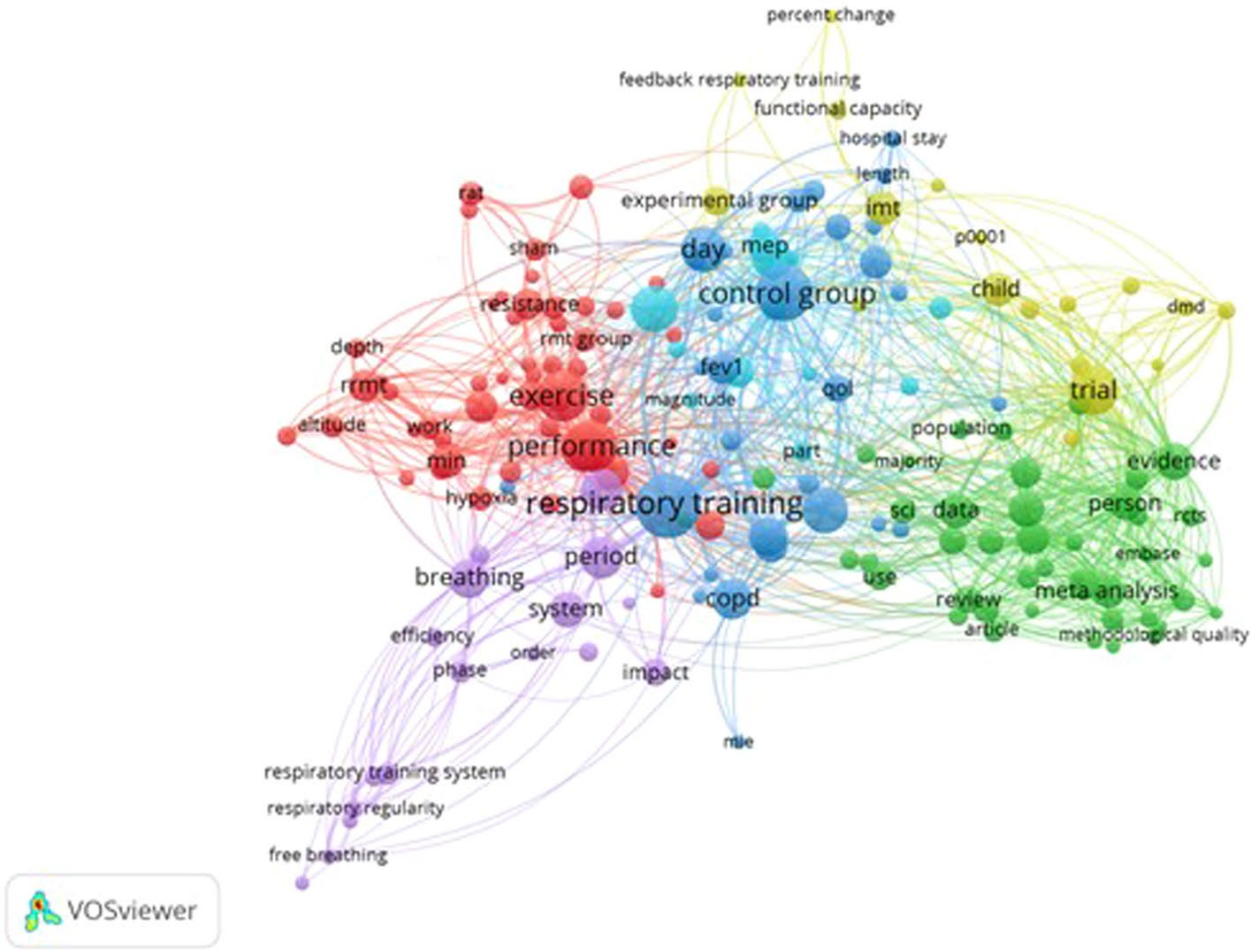
Visualisation map of the term co-occurrence network based on title and abstract fields. Full counting, minimum number of occurrences of a keyword: 10

Meanwhile, Fig. 4 shows the visualisation map of the term co-occurrence based on title and abstract fields. This figure represents the relatedness of the terms obtained across the title and abstract of all literatures. Figure 4 was grouped into five clusters, indicated by five different colours, after a minimum number of ten occurrences of a term was set up.

### RMT studies among people with SCI

Further screening of the 367 total literature search of the RMT retrieved mansucripts in this study focused on only people with SCI as the RMT recepients resulted in 36 papers.

### Intervention

**Fig. 5:**
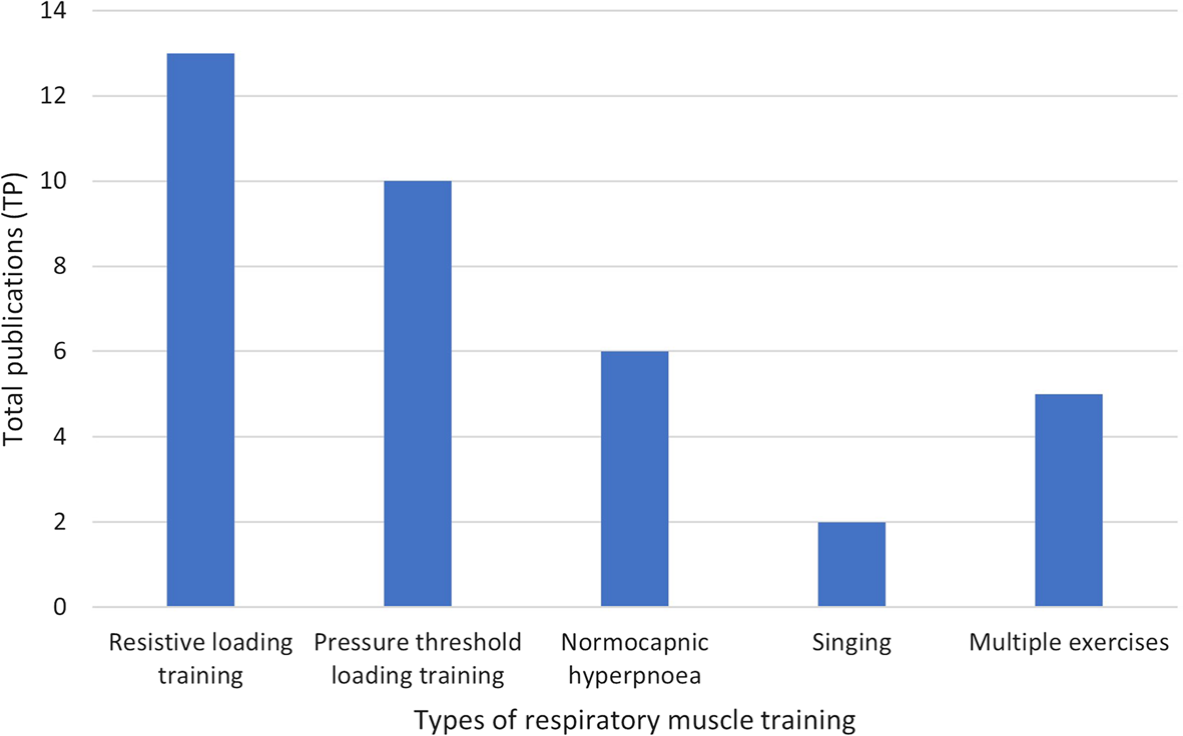
Five types of RMT utilised in studies among people with SCI

Figure 5 shows the five different types of RMT techniques utilized by researchers across all 36 publications. These techniques include the resistive loading training, pressure threshold loading training, normocapnic hyperpnoea, singing therapy and multiple or combined exercises. In resistive training, the participants inhaled or exhaled through a variable size or diameter of hole that acts as a resistor. This technique limit the available airflow, therefore, increases the ventilatory load.

In pressure threshold training, the participants inhaled or exhaled to overcome a spring-loaded valve with sufficient force, therefore, enable airflow. In normocapnic hyperpnoea, participants filled and emptied a bag connected to a mouthpiece and tube completely with each breath. This bag was set at 30% to 40% of participants’ vital capacity. In certain publication, the researchers utilized multiple training, and sometimes combined exercises on their participants. For instance, Mueller et al. implemented both resistive loading training and normocapnic hyperpnoea to compare their effectiveness [22]. Kim et al. integrated RMT with abdominal drawing-in maneuver to activate the transverse muscle during training [23]. While, in specific cases, the researchers conducted the RMT by singing therapy.

### Intervention tools and outcome measures

**Fig. 6:**
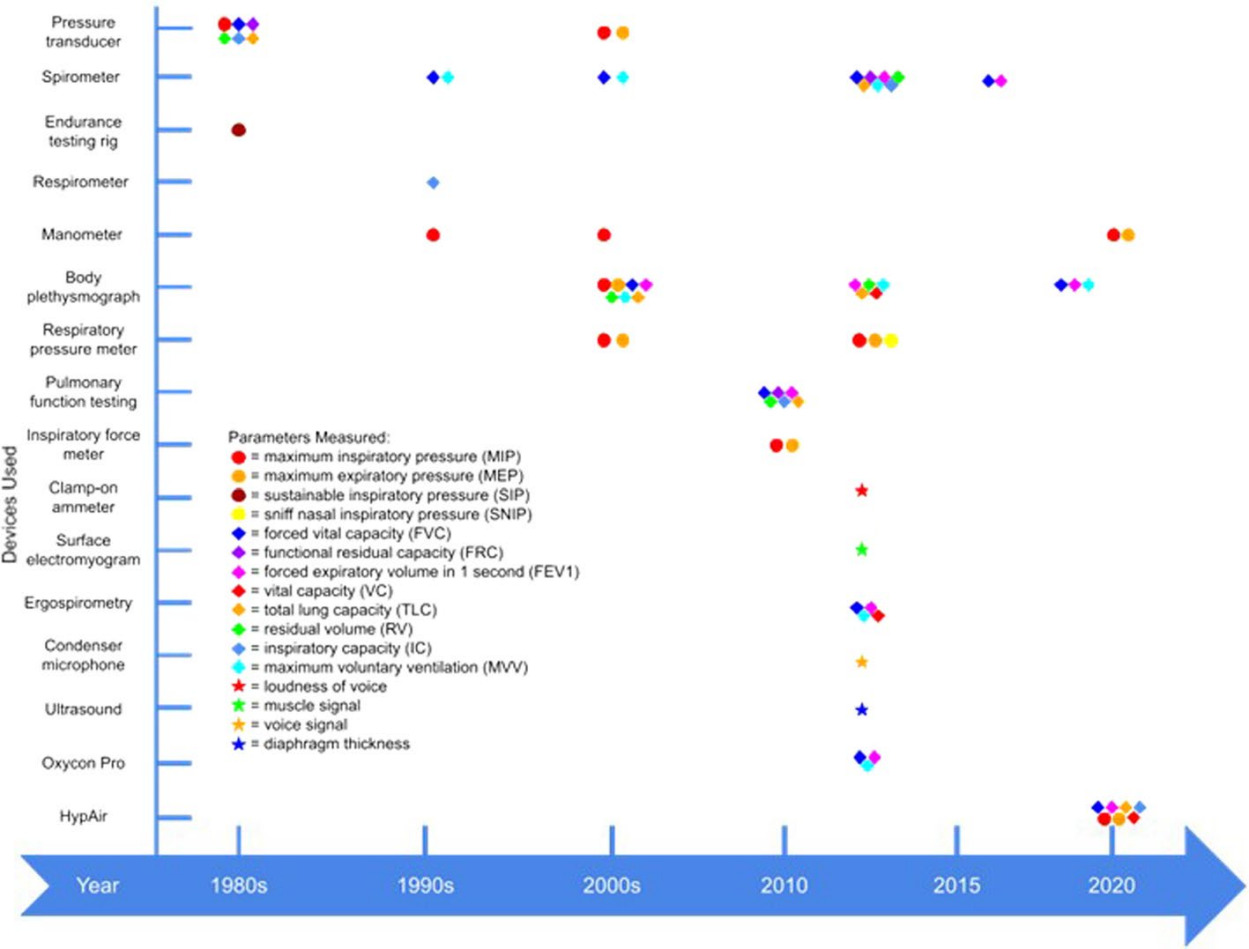
Timeline of devices used in measuring main outcomes in RCT studies among people with SCI. circle = respiratory muscle strength (main outcome), diamond = pulmonary function, star = others

Out of the 36 publications identified in this section, 16 of them were identified as randomized-controlled trials (RCT). These RCTs were analysed according to the outcome measures in the pulmonary function, respiratory muscle strength and muscle activity. Table 10 summarizes the interventions, main outcome measures, and every devices used in RCT studies among people with SCI. Meanwhile, Fig. 6 illustrates the timeline of the devices used in measuring the main outcome measures or parameters in these RCT studies throughout 6 decades.

**Table 10:** List of all the devices used during the RMT intervention in RCT studies among people with SCI

Author, year	Intervention	Device	Main outcome measures	Device
Loveridge et al., (1989) [16]	Resistive loading training	Inspiratory resistor	Respiratory muscle strength	Validyne pressure transducer
			Ventilation timing	Mercury-in-rubber strain gauge^a^ Microcomputer and a polygraph^b^
			Pulmonary function	Ventilatory muscle endurance testing rig A single lead ECG and end tidal CO_2_^c^
Derrickson et al., (1992) [17]	Resistive loading training	Inspiratory muscle trainer^d^	Pulmonary function	Volume displacement spirometer^e^ Respirometer^f^
			Respiratory muscle strength	Manometer^g^
Liaw et al., (2000) [24]	Resistive loading training	Inspiratory muscle trainer^d^	Respiratory muscle strength	Respiratory pressure meter^h^
			Lung volumes	Body plethysmography^i^
			Pulmonary function	
Van Houtte et al., (2008) [25]	Normocapnic hyperpnoea training	Lab-developed normocapnic device	Respiratory muscle strength	Electronic pressure transducer
			Pulmonary function	Portable spirometer^j^
Litchke et al., (2008) [26]	Resistive loading training	Concurrent flow respiratory device^k^	Respiratory muscle strength	Manometer^l^
			Pulmonary function	Spirometer^m^
			Oxygen uptake	Heart rate monitor^n^ Air analyser^o^
Roth et al., (2010) [27]	Resistive loading training	High pressure inspiratory force meter^q^	Respiratory muscle strength	High pressure inspiratory force meter^q^
			Pulmonary function	Pulmonary function testing^r^
(Mueller et al., 2013) [22]	Resistive loading training	Inspiratory threshold trainer^s^	Respiratory muscle strength	Respiratory pressure meter^t^
			Pulmonary function	Body plethysmography^u^
	Normocapnic hyperpnoea training	Respiratory endurance test^p^		
			Loudness of voice	Clamp-on ammeter^v^
Tamplin et al., (2013) [28]	Singing training	Karaoke game^w^	Respiratory muscle strength	Respiratory pressure meter^x^
			Pulmonary function	Spirometer^y^
			Lung volume	Spirometer with a helium analyzer^z^
			Voice signal	Condenser microphone^ab^
			Muscle signal	Surface electromyography
Postma et al., (2014) [29]	Pressure threshold loading training	Inspiratory threshold trainer^ac^	Respiratory muscle strength	Respiratory pressure meter^ad^
			Pulmonary function	Spirometer^ae^
			Lung volumes	
West et al., (2014) [30]	Pressure threshold loading training	Pressure threshold device^af^	Respiratory muscle strength	Respiratory pressure meter^h^
			Pulmonary function and oxygen uptake	Stationary automated metabolic gas analysis system^ag^
			Diaphragm thickness	B-mode two-dimensional ultra- sound^ah^
Fischer et al., (2014) [31]	Normocapnic hyperpnoea training	Respiratory endurance test^p^	Pulmonary function	Ergospirometric device^ai^
Kim et al., (2017) [23]	Integrated training	Incentive spirometer^aj^	Pulmonary function	Computerized spirometer^ak^
Xi et al., (2019) [32]	Normocapnic hyperpnoea training	Respiratory endurance test^p^	Pulmonary function	Body plethysmography^u^
Soumyashree and Kaur, (2020) [33]	Pressure threshold loading training	Inspiratory muscle trainer^al^	Respiratory muscle strength	Capsule sensing pressure gauge (CSPG-V) manometer^am^
Boswell-Ruys et al., (2020) [34]	Pressure threshold loading training	Inspiratory threshold trainer^ac^	Pulmonary function	Lung function machine^an^
Litchke et al., (2010) [35]	Resistive loading training	Concurrent flow respiratory device^k^	Pulmonary function	Spirometer^m^
	Pressure threshold loading training	Concurrent pressure threshold resistance device^ao^	Respiratory muscle strength	Manometer^l^

N/M = not mentioned, the small letters in subscript at the last column indicates the suppliers listed in Appendix 1

### Vital capacity (VC)

Five RCT studies had measured VC of participants post-RMT in both intervention and control groups [22, 24, 28, 31, 34]. In total, 74 participants were recruited in the intervention group, while 66 participants were in the control group. Liaw et al. reported significant difference (*P* < 0.05) between pre- and post-RMT within both intervention and control groups [24].

### Maximal inspiratory pressure (MIP)

From 12 RCT studies that measured MIP, a total of 135 participants were recruited each in intervention and control groups [16, 17, 22, 24–30, 33, 34]. Five studies recorded significant difference (*P* < 0.05) between pre- and post-RMT within intervention group [17, 24, 25, 27, 33].

Derrickson et al., Liaw et al. and Soumyashree and Kaur also reported significant difference (*P* < 0.05) within control group [17, 24, 33]. Six studies also recorded significant difference (*P* < 0.05) in post- RMT values between intervention and control groups [25, 26, 29, 30, 33, 34].

### Maximal expiratory pressure (MEP)

Nine RCT studies had measured MEP in their publications with a total of 129 and 119 participants in their intervention and control groups, respectively [22, 24, 25, 27–30, 33, 34]. Four studies published significant difference (*P* < 0.05) between pre- and post-RMT within intervention group [24, 25, 27, 33]. Liaw et al. also reported significant difference (*P* < 0.05) within control group [24]. Meanwhile, van Houtte et al. and Roth et al. recorded significant difference (*P* < 0.05) in post-RMT values between the intervention and control groups [25, 27].

### Forced expiratory volume in 1 second (FEV***1***)

With a total of 147 and 139 participants in intervention and control groups, respectively, ten RCT studies had measured FEV_1_ in their publications [22–24, 27–32, 34]. Three publications reported significant difference (*P* < 0.05) between pre- and post-RMT within intervention group [23, 24, 27]. Liaw et al. and Roth et al. also recorded significant difference (*P* < 0.05) within control group [24, 27]. Meanwhile, Kim et al. and Xi et al. recorded significant difference (*P* < 0.05) in post- RMT values between intervention and control groups [23, 32].

### Forced vital capacity (FVC)

From 11 RCT studies that measured FVC, a total of 144 participants were recruited each in intervention and control groups [17, 23–25, 27–32, 34]. Five publications found significant difference (*P* < 0.05) between pre- and post-RMT within intervention group [17, 23–25, 27]. Three publications also reported significant difference (*P* < 0.05) within control groups [17, 24, 27]. Meanwhile, several publications found significant difference (*P* < 0.05) in post-RMT values between intervention and control groups [23, 25, 32, 34].

### Maximal voluntary ventilation (MVV)

Eight RCT studies had measured MVV in their publications with a total of 72 and 66 participants in their intervention and control groups, respectively [17, 22, 25, 29–32, 35]. Three publications found significant difference (*P* < 0.05) between pre- and post-RMT within intervention group [17, 25, 30]. Derrickson et al. and West et al. also reported significant difference (*P* < 0.05) within control group [17, 30]. Meanwhile, van Houtte et al. and Xi et al. recorded significant difference (*P* < 0.05) in post- RMT values between intervention and control groups [25, 32].

### Respiratory muscle activity

Out of the 16 RCTs, only Tamplin et al. measured the respiratory muscle activity during the RMT intervention. Using surface electromyography (EMG), the accessory respiratory muscles activity were measured during singing therapy [28]. There was significant difference in pre-post change in respiratory muscle activation from speech to singing between groups (*P* = 0.015). In intervention group, there was significant difference in pre-post change in respiratory muscle activation from speech to singing (*P* = 0.007).

## Discussion

### Bibliometric analysis

Research on RMT has been steadily increasing for the last 6 decades (Table 4) (Fig. 1). As shown in Table 4 and Fig. 1, publications from authors of medicine background dominates the research on this topic in the same period of time. The first reported research related to RMT, back in 1961, also came from the medicine background. Scopus also generated health professionals, neuroscience and biochemistry as one of the top subject areas over the last 60 years. Other than medicine, nursing and health professionals were the earliest subject areas published in this topic, dated back in 1974.

Since then, many other subject areas started to appear in publishing this topic, including engineering, computer science, immunology, pharmacology, and social sciences. One publication from engineering started to emerge from 1992. Since then, a total of 14 literatures related to engineering has been published. The year with the highest number of publications with engineering background were in 2004 and 2014, with four and three publications, respectively. Besides, chemical engineering was also generated by the Scopus database as early as in 2008. Since then, four literatures related to chemical engineering has been published. While medicine continues to dominate the research on RMT, this topic also continues to attract researchers and publications from other areas, especially over the last 10 years.

Figure 2 shows that majority of the publications were originated from the United States and Europe. A bibliometric analysis of global research on respiratory medicine also reported the same output [36]. From Table 6, it is worth to note that some of the authors were affiliated with highly integrative environments. For instance, in Zurich Center for Integrative Human Physiology, the principal investigators of the postgraduate program have different research background, including engineering. In Department of Head and Neck Surgery and Communication Sciences, Duke University School of Medicine, aside from medicine, one of the programs offered in the department was communication sciences. This program includes audiologists and speech pathologists. Similarly with Center for Research and Education in Special Environments in University at Buffalo, The State University of New York, their principal investigators also came from a wide range of background, including public health professionals, engineering and applied sciences.

It is also interesting to note that the top two of the authors in Table 6, namely Pendergast and Lundgren, had collaborated multiple times and published several publications in this topic over the last 15 years. They have published a total of at least eight literatures associated with RMT since 2006. Similarly with Crisp, Jones and Kravitz. These authors had collaborated in this topic in a total of five publications since 2014. Each of these authors were affiliated with a center and department that involve researchers from multiple backgrounds, including medicine and engineering. Therefore, the nature of multidisciplinary in RMT research can be traced as early as 2006, and have been increasing ever since. Based on Table 9, we can also see source title that affiliates with subject area other than medicine, such as Journal of Voice and Medical Physics. This shows that many researchers and publications from different backgrounds have started to show an interest in this topic.

Continuing the multidisciplinary research in RMT in the future may benefits the targeted population. As more researchers from different field of studies collaborated, more things can be achieved and solved. Instead of focusing only on new evidence, multidisciplinary research may use those evidences to the next level and produce efficient service to the targeted community. For instance, such collaboration may leads to the advancement of current technology and intervention in monitoring respiratory muscle performance during RMT. Multidisciplinary research may also help in the innovation of future technological advancement in artificial lungs in general to support respiratory and gas exchange.

### RMT studies among people with SCI

From Fig. 5, researchers mainly focused and utilised resistive loading and pressure threshold loading training as their RMT interventions. Singing training has been introduced in the SCI population as early as 2011 by Tamplin et al. Since then, Tamplin et al. had also published an RCT study among people with SCI by incorporating singing as RMT in the intervention group.

From the RCT studies summarized in Table 10, multidisciplinary study was shown the most in 2013. Some of the devices were used by Mueller et al. and Tamplin et al. to monitor and measure the participants’ voice and muscle signals, including condenser microphone and surface electromyography [22, 28]. Some of the authors in these publications were also affiliated from various backgrounds, such as Institute of Sports Medicine, Swiss Paraplegic Centre, Nottwill, Switzerland and Voice Analysis Clinic, Austin Health, Melbourne, Australia.

A majority of the RCT studies in Table 10 reported respiratory muscle strength and pulmonary function as their main outcome measures. These parameters include MIP, MEP, VC, FVC, FEV_1_ and MVV. Various kind of spirometers have been utilised by the researchers to seek out these parameters. The results obtained shows that it is more frequent to witness significant difference between pre- and post-RMT within intervention group compared to control group. Plus, some studies also recorded significant difference in post-RMT values between intervention and control groups. Several other outcome measures were also measured in these RCT studies, including lung volumes, diaphragm thickness, oxygen uptake, voice signal and respiratory muscles signal.

Before 2014, every RCT studies on RMT on people with SCI measured at least two main outcome measures in their studies. Interestingly, since 2014, every RCT studies focuses on a singular main outcome measures, either pulmonary function or respiratory muscle strength. It may seemed that as of late, researchers tends to focus on one angle of parameters, and leverage on the data to investigate the effectiveness of RMT and compare the parameters before and after RMT.

The timeline in Fig. 6 shows the utilisation trend of devices in measuring main parameters in RMT studies among people with SCI. Some devices were used to measure several pulmonary function and lung volumes outcomes at once, such as pressure transducer, spirometer and body plethysmograph. In 2014 and 2020, researchers used advanced and compact devices that can measure a wide range of respiratory muscle strength and pulmonary function parameters, such as Oxycon Pro and HypAir. Spirometer has steadily been used and featured in RMT studies throughout the decades, including in the 1990s. In the 2010s, researchers started to implement specific devices to measure specified parameters. For example, Mueller et al. utilised a clamp-on ammeter to measure the loudness of voice [22]. Meanwhile, Tamplin et al. implemented a condenser microphone and surface electromyography to record the voice and muscle signal during singing [28].

## Conclusion

To conclude, there has been a constant increase number of publications on RMT in various populations over the last 6 decades. Publications from medicine background has been dominating this research since it started back in the early 1960s. Since then, many more subject areas had emerged to conduct and publish research on RMT, including engineering, biochemistry and computer science. There has also been a trend of multidisciplinary study and collaboration between authors of different backgrounds and expertise, especially since 2006. Source title that affiliates with non-medicine background also appeared to be one of the most active source title to publish research on RMT.

Meanwhile, subanalysis on RMT studies on people with SCI had shown that most publications implemented conventional RMT such as resistive and pressure threshold training. Others had either introduced a new intervention such as singing, or implemented combined training among their participants. Prior to 2017, researchers reported multiple outcome measures in their publications, such as diaphragm thickness, oxygen uptake, voice signal and respiratory muscles signal. Since then, researchers tends to focus on one angle of outcome measures, either respiratory muscle strength or pulmonary function only. Devices used in capturing the main outcome measures also became more advanced and compact as the years go by. Researchers also started to incorporate specific devices to measure a more specific parameters, such as muscle signal and loudness of voice. In general, there has been an improvement in respiratory muscle strength and pulmonary function parameters among people with SCI after RMT.

### RMT study impact

As the COVID-19 pandemic and air pollutions continue to impact our society, the need for innovations to treat respiratory issues has never been so high. Researchers may take advantage on artificial intelligence in developing a more accurate breath measuring algorithms. Smart inhalers may also improve the way vital informations relayed to doctors. Wearable analytics are also important to ensure better adherence of patients to the treatment. Therefore, to encourage such collaboration in the future, grant providers should actively look out for non-conventional recipients. By doing so, research will be more impactful and beneficial in improving acute and chronic respiratory conditions that impact more than one billion people every year [37]. Policy makers can also utilise the new evidence from research to create a safer and better lifestyle in the community. For example, smokefree policies in public area has been one of the successful measures not only to improve health and air quality, but also to reduce secondhand smoke exposures.

### Limitation of the study

The subject area categorization was directly obtained from the Scopus database. Therefore, the subject area represented the background of the journals. Scopus also compiled both conference proceeding (full papers) and abstract as part of conference paper in document type (Table 1). However, in source type (Table 2), abstract was compiled together with journal. Hence, the count between conference paper and conference proceeding in Tables 1 and 2, respectively, was not synchronized. Some keywords such as “ventilatory muscle training”, “inspiratory muscle training” and “expiratory muscle training” were not included during the literature search process. This could affect the number of literatures included in this review.

## Methods

### Literature search strategy

**Fig. 7:**
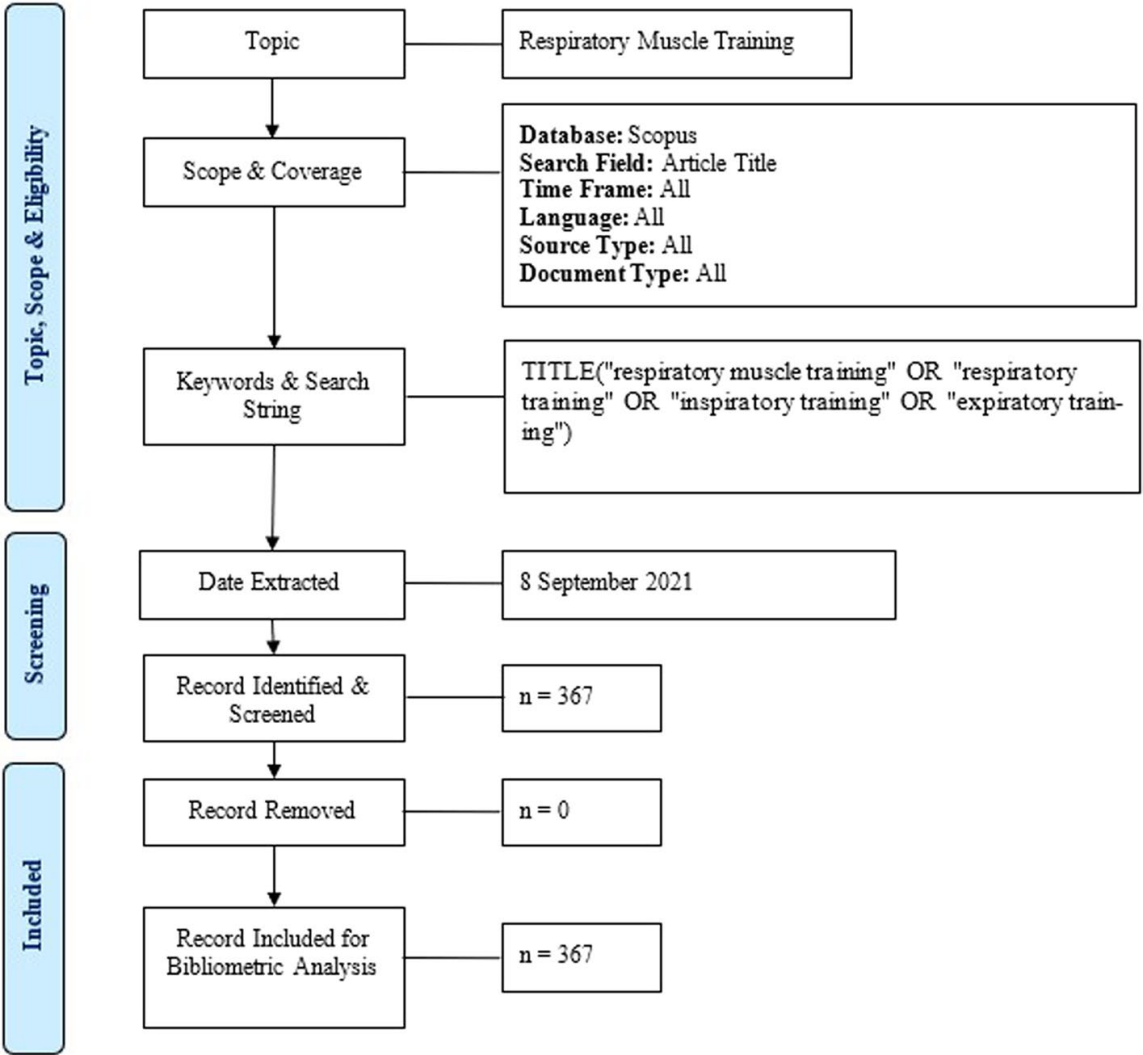
Flow diagram of the search strategy [21]

The literature search was conducted in the electronic database Scopus on 8th September 2021. The research protocol for this bibliometric analysis is summarized in a flow diagram in Fig. 7. To get more accurate literatures for this analysis, the search was done within the article title only. Considering the inadequate effort on the bibliometric analysis of respiratory muscle training, the literature search was done on all time frame, languages, source type and document type.

Once gathered, these documents were exported in comma-separated values (.csv) and research formatted systems (.ris) files. These files were used for the bibliometric visualisation map and citation metrics analyses. Meanwhile, the refine value of the search result was exported into a separate comma-separated values (.csv) file for basic bibliometric analysis.

### Eligibility criteria

All articles related to respiratory muscle training from Scopus was included in this review. Other than “respiratory muscle training”, several other keywords were also included during the literature search process. This includes “respiratory training”, “inspiratory training” and “expiratory training”.

### Data extraction and analysis

The data extraction from Scopus database was carried out on the same day as the literature search; 8^th^ September 2021. Microsoft Excel, Harzing’s Publish or Perish, and VOSviewer softwares were used for the data analyses in this bibliometric review. Some basic bibliometric results and document profiles were analysed from the exported refine value of the search result in Excel file. This includes the year, author, subject area, document type, source title, source type, affiliation, country, and language of all documents extracted.

To gather the research trends and citation metrics, the Harzing’s Publish or Perish software was utilised. This includes the number of cited papers (NCP), total citations (TC), average citations per publications (C/P), average citations per cited publications (C/CP), h-index and g-index. Some information were also gained manually from the Scopus database itself, such as the scientific journal ranking (SJR), source normalized impact per paper (SNIP), cite score, publisher, affiliation and country of origin. In certain situation, the filter feature in the Scopus database was used to obtain a more specific result for the bibliometric analysis. For example, the trends of publications by year and subject area was obtained by limiting the search result in desired year and subject area.

Meanwhile, the VOSviewer software was specifically used to capture maps based on both bibliometric and text data. In the map based on bibliometric data, keyword co-occurrence analysis was done. The keywords were extracted from both the author and index keywords. In the map based on text data, term co-occurrence analysis was done. The terms were extracted from both the title and abstract fields. The relatedness of the keywords or terms was determined based on the number of the documents in which they occur together. The bigger the keyword or term represented in the map, the higher the number of the keyword co-occurrence across all literatures.

### Subgroup analysis of RMT studies involving people with SCI

The authors are interested to further analyse studies conducted in people with SCI. Thus, a subanalysis on this population is carried out. The authors extracted all SCI-related studies and describe the progress, trend, types of interventions and outcomes used.

## Data Availability

The datasets used and/or analysed during the current study are available from the corresponding author on reasonable request.

## APPENDIX 1: SUPPLIERS (as cited in Table 10)

^a^Hewlett Packard, Model 9816.

^b^Model 2600S, Gould Recorder.

^c^Perkin-Elmer, Medical Gas Analyzer, Model 1100.

^d^Diemolding Healthcare Division, Canastota, NY 13,032.

^e^Warren E Collins Inc, 220 Wood Rd, Braintree, MA 02,184.

^f^Fraser Harlake, 145 Midcounty Dr, Orchard Park, NY 14,127.

^g^Boehringer, 4427 Parkview Dr, Wynnewood, PA 19,096.

^h^Micro Medical Limited, PO Box 6, Rochester, Kent, MEI 2AZ, England.

^i^SensorMedics 280D; SensorMedics Corporation, 1630 S State College Blvd. Anaheim, CA 92,806.

^j^Spirobank, Medical International Research, Italy.

^k^Expand-a-Lung Inc, Miami, FL.

^l^Instrumentation Industries, Bethal Park, PA.

^m^EasyOne Spirometer, NDD Medical Technologies, Andover, MD.

^n^Polar Vantage XL telemetric HR monitor, Stamford, CT.

^o^PARVO Medics metabolic analyzer, Salt Lake City, UT.

^p^Spirotiger®, SpiroTiger Medical, Idiag AG, Fehraltorf, Switzerland.

^q^Boehringer Labs Inc, PO Box 870, Norristown, PA 19,404.

^r^Cybermedics Inc, 1341 Cannon St, Louisville, CO 80,027.

^s^Respifit S®, Eumedics gmbH, Purkersdorf, Austria.

^t^Micro RPM, Micro Medical, Hoechberg, Germany.

^u^Master Screen®Body, Viasys Healthcare GmbH, Hoechberg, Germany.

^v^Voltcraft 320, Conrad Electronic SE, Hirschau, Germany.

^w^SingStar for PlayStation 2; Sony, 550 Madison Ave, New York, NY, 10,022.

^x^EasyOne Spirometer, OPS Medical, LLC, 8055 Ritchie Hwy, Ste 103, Pasadena, MD 21,122.

^y^MicroRPM, Compumedics Sleep Pty Ltd, 1 Marine Parade, Abbotsford, Victoria, 3067, Australia.

^z^P K Morgan M8 rolling seal spirometer, Micro Medical Ltd, PO Box 6, Rochester, Kent ME1 2AZ, UK.

^ab^Ono Sokki MI-1211 Type 1, VIPAC Engineers &Scientists Ltd, Victorian Technology Centre, 275 Normanby Rd, Port Melbourne, Victoria, 3207, Australia.

^ac^Threshold IMT, Respironics Inc, Parsippany, New Jersey.

^ad^MicroRPM, CareFusion, Basingstoke, United Kingdom.

^ae^Oxycon Delta, CareFusion, Hoechberg, Germany.

^af^POWERbreathe Plus, HaB International Ltd, Southam, UK.

^ag^Oxycon Pro, Jaeger, Höchberg, Germany.

^ah^Esaote Megas GPX, Imotek, Somersham, UK; 10 MHz linear array transducer.

^ai^QUARK PFT; Cosmed, Rome, Italy.

^aj^Tri-ball Incentive Spirometer 600–1200 cc, Ark Therapeutic, Lugoff, USA.

^ak^Chestgraph HI-101, Chest MI Inc., Tokyo, Japan.

^al^Philips Threshold® IMT Inspiratory Muscle Trainer.

^am^Gauges Bourdon (I) pvt. Ltd, India.

^an^Hyp’air, Belgium.

^ao^Powerlung inc., Houston, TX.

## Abbreviations

C/CP: Average citations per cited publications
C/P: Average citations per publications
FEV_1_: Forced expiratory volume in 1s
FVC: Forced vital capacity
MIP: Maximal inspiratory pressure
MEP: Maximal expiratory pressure
MVV: Maximal voluntary ventilation
NCP: Number of cited papers
RCT: Randomized-controlled trials
RMT: Respiratory muscle training
SJR: Scientific journal ranking
SNIP: Source normalized impact per paper
SCI: Spinal cord injury
TC: Total citations
VC: Vital capacity
